# Antimicrobial resistance and molecular characterization of *bla*_CTX-M_-producing *Escherichia coli* isolated from broiler chickens in Peru

**DOI:** 10.1371/journal.pone.0360681

**Published:** 2026-10-13

**Authors:** Edgar Gonzales-Escalante, Arturo Gonzales-Rodriguez

**Affiliations:** Facultad de Medicina Humana, Universidad de Piura, Lima, Peru; The University of Tennessee Knoxville College of Veterinary Medicine, UNITED STATES OF AMERICA

## Abstract

The misuse and overuse of antimicrobials in humans and animals contribute to the selection and dissemination of antimicrobial-resistant (AMR) bacteria. In animal production systems, antimicrobial use is a key driver of the emergence and spread of antimicrobial-resistant bacteria across humans, animals, and the environment within a One Health framework. A total of 300 cloacal swabs were collected from apparently healthy broiler chickens at two poultry collection centers in Lima, Peru. ESBL-producing *Escherichia coli* were isolated on CHROMagar™ ESBL and identified by conventional biochemical tests. Antimicrobial susceptibility testing was performed using the disk diffusion method according to CLSI guidelines, and ESBL production was phenotypically confirmed. CTX-M groups were detected by qPCR, and whole-genome sequencing (WGS) was performed on selected isolates. Seventy-nine ESBL-producing *E. coli* isolates were recovered (26.3%; 95% CI: 21.4–31.6). All isolates exhibited multidrug-resistant phenotypes. CTX-M group 1 was detected in 87.3% of isolates and group 9 in 12.7%. WGS analysis identified *bla*_CTX-M-55_, *bla*_CTX-M-65_, and *bla*_CTX-M-3_ distributed across multiple sequence types. Phylogenetic analysis revealed the presence of both closely related clonal clusters and genetically diverse isolates. These findings suggest that poultry production systems may serve as potential reservoirs of multidrug-resistant ESBL-producing *E. coli* in Peru. Given the study design and limited genomic sampling, the results should be interpreted as preliminary and highlight the need for expanded surveillance within a One Health framework.

## Introduction

Over the past decade, chicken meat has been considered the leading source of animal protein globally, attributable to its affordability, efficient production systems, and high nutritional profile [1]. Peru records one of the largest per capita consumption rates of chicken meat in South America. Poultry farming represents the country’s primary food animal production [2]. In animal production systems, the unjustified use and overuse of antimicrobials contribute significantly to the selection and dissemination of resistant bacterial populations [3–5]. Antimicrobials are frequently administered not only for therapeutic purposes but also for prophylactic and metaphylactic use. In some regions, their use as growth promoters has historically contributed to the emergence and persistence of resistant bacteria, although regulatory frameworks vary across countries [3–5]. Poultry production represents one of the largest sectors of antimicrobial consumption in food-producing animals worldwide [4]. Consequently, poultry-associated bacterial populations are considered important reservoirs of antimicrobial resistance. Transmission of resistant bacteria from poultry to humans may occur through direct contact, environmental exposure, or the consumption of contaminated food products [4,5]. Peru is a middle-income country struggling with AMR in the livestock sector, given the widespread misuse of antibiotics, along with farmers’ poor traditional knowledge regarding antibiotics use [2]. In particular, an increase of 160% in livestock antimicrobial use was predicted between 2015 and 2030 [6]. The poultry sector is the largest in the country, with 191.7 million chickens contributing to 24% of the national Gross Livestock Production Value (GLVP) in 2023 (USD ~ 2.65 billion) [7].

Among these resistant bacteria, *E. coli* is commonly used as an indicator organism for monitoring AMR due to its ubiquity and ability to acquire and transfer resistance determinants [8]. Of particular concern is resistance to third-generation cephalosporins mediated by extended-spectrum β-lactamases (ESBLs), and plasmid-mediated AmpC β-lactamase (pAmpC) enzymes, which has increased markedly over the past two decades [9]. Within this group, the CTX-M enzymes represent the most prevalent and globally disseminated ESBL family, frequently detected in both human clinical isolates and animal reservoirs [9,10].

In Latin America, and particularly in Peru, available data on ESBL-producing *E. coli* in poultry remain limited, despite increasing evidence of antimicrobial resistance in food-producing animals [11–14]. Therefore, this study aimed to investigate the antimicrobial resistance profiles and molecular characteristics of *bla*_CTX-M_-type ESBL-producing *E. coli* isolated from broiler chickens in Peru, contributing to the understanding of their potential role in the dissemination of antimicrobial resistance within a One Health framework.

## Materials and methods

### Study setting and sampling

Sampling was conducted at two commercial poultry collection centers located in Lima, Peru. These centers function as aggregation points where broiler chickens from multiple farms are gathered prior to distribution to markets. Therefore, the sampled population represents a mixed-source population rather than individual farms. A total of 300 cloacal swabs were collected from apparently healthy broiler chickens, in November 2022 (150 from each center). Sampling was performed using a convenience approach due to logistical constraints. The total population size of chickens passing through these centers was not available; therefore, the findings should be interpreted as representative of the sampled population rather than the broader poultry production system.

### Microbiological analysis

Swabs were transported in Cary–Blair transport medium to the Microbiology Laboratory of the Faculty of Human Medicine, University of Piura. For presumptive isolation of *E. coli*, samples were inoculated onto CHROMagar™ ESBL (Paris, France) and incubated at 35 °C for 24 h. Colonies with characteristic red–pink coloration were selected as presumptive ESBL-producing *E. coli*. Species identification was confirmed by conventional biochemical testing, including triple sugar iron agar (TSI), citrate utilization, urease production, indole production, methyl red test, and motility assays, as previously described [8].

### Antimicrobial susceptibility testing

Antimicrobial susceptibility testing was performed using the disk diffusion method according to CLSI guidelines (2025) [15]. The following antimicrobials were tested: cefotaxime (30 µg), ceftazidime (30 µg), cefepime (30 µg), cefoxitin (30 µg), imipenem (10 µg), meropenem (10 µg), ciprofloxacin (5 µg), trimethoprim–sulfamethoxazole (1.25/23.75 µg), chloramphenicol (30 µg), fosfomycin (200 µg), amikacin (30 µg), and gentamicin (10 µg) (Bioanalyse®, Ankara, Turkey). Quality control was performed using *Escherichia coli* ATCC 25922. Multidrug resistance (MDR) was defined as non-susceptibility to at least one agent in three or more antimicrobial classes. Phenotypic confirmation of ESBL production was performed using the double-disk synergy test (Jarlier method) as previously described [16].

### Molecular identification of ESBL-producing *E. coli*

Genotypic detection of CTX-M groups was performed by real-time PCR (qPCR) following DNA extraction and purification using the GeneJET Genomic DNA Purification Kit (Thermo Scientific, Lithuania), according to the manufacturer’s instructions and using a validated protocol [10] on a Rotor-Gene Q (QIAGEN, Hilden, Germany).

### Genomic analysis

Nine isolates were selected for whole-genome sequencing (WGS) based on the proportional representation of sampling origin and the diversity of resistance mechanisms. Genomic sequencing was performed using Illumina MiSeq technology with paired-end reads (2 × 250 bp). De novo genome assembly was conducted using Unicycler v0.4.8 [17]. Genome assemblies were annotated using Prokka v1.14.6 [18] and manually curated.

Genomic characterization was performed using bioinformatic tools available through the Center for Genomic Epidemiology platform (https://www.genomicepidemiology.org/) and ClermonTyping (https://clermontyping.iame-research.center/). Multilocus sequence typing (MLST) was determined using MLSTfinder; antimicrobial resistance genes were identified using ResFinder; virulence-associated genes were detected using VirulenceFinder; phylogenetic grouping was assigned using the ClermonTyping scheme; and flagellar (H) and lipopolysaccharide (O) antigen loci were determined using SeroTypeFinder. Default parameters were used for all bioinformatic tools unless otherwise specified.

### Phylogenetic tree

Genetic relationships among isolates were inferred based on single-nucleotide polymorphism (SNP) analysis. SNPs were identified and extracted using SNP-sites v2.5.1 [19], and a maximum-likelihood phylogenetic tree was constructed using IQ-TREE v2.5.1 [20], applying the best-fit substitution model automatically selected by the software and 1,000 bootstrap replicates to assess branch support. Phylogenetic tree visualization and interactive exploration were performed using Microreact [21].

### Statistical analysis

A descriptive and inferential statistical analysis was performed. The chi-square test (χ²) was used to assess statistically significant differences in the antimicrobial susceptibility profiles of ESBL-producing *E. coli* isolates between the two collection centers, with statistical significance defined as p < 0.05.

### Ethical approval

This study was approved by the Institutional Research Ethics Committee of the University of Piura (approval code: PREMED11202232; approval date: November 2022). Access to the commercial poultry farms was granted by the farm owners and administrators. Representatives of each participating center signed a Material Transfer Agreement (MTA) authorizing the use of samples exclusively for research purposes. No specific governmental permits were required for sample collection, as the study was conducted on privately owned farms and did not involve protected areas or endangered species. Animal subjects (chickens from commercial poultry farms) were sampled exclusively by cloacal swab collection, and no invasive or harmful procedures were performed during animal handling.

## Results

### Isolation of ESBL-producing *E. coli*

A total of 79 ESBL-producing *E. coli* isolates were recovered from 300 cloacal swabs (26.3%; 95% CI: 21.4–31.6), including 37 isolates from center A and 42 from center B.

### Antimicrobial susceptibility profile

The antimicrobial susceptibility profiles of the 79 ESBL-producing *E. coli* isolates are shown in Fig 1. High levels of resistance to third-generation cephalosporins were observed in isolates from both collection centers, with 100% resistance to cefotaxime. Non-susceptibility (resistant + intermediate) rates were 51/79 (64%) for ceftazidime and 69/79 (87%) for cefepime**.**

**Fig 1:**
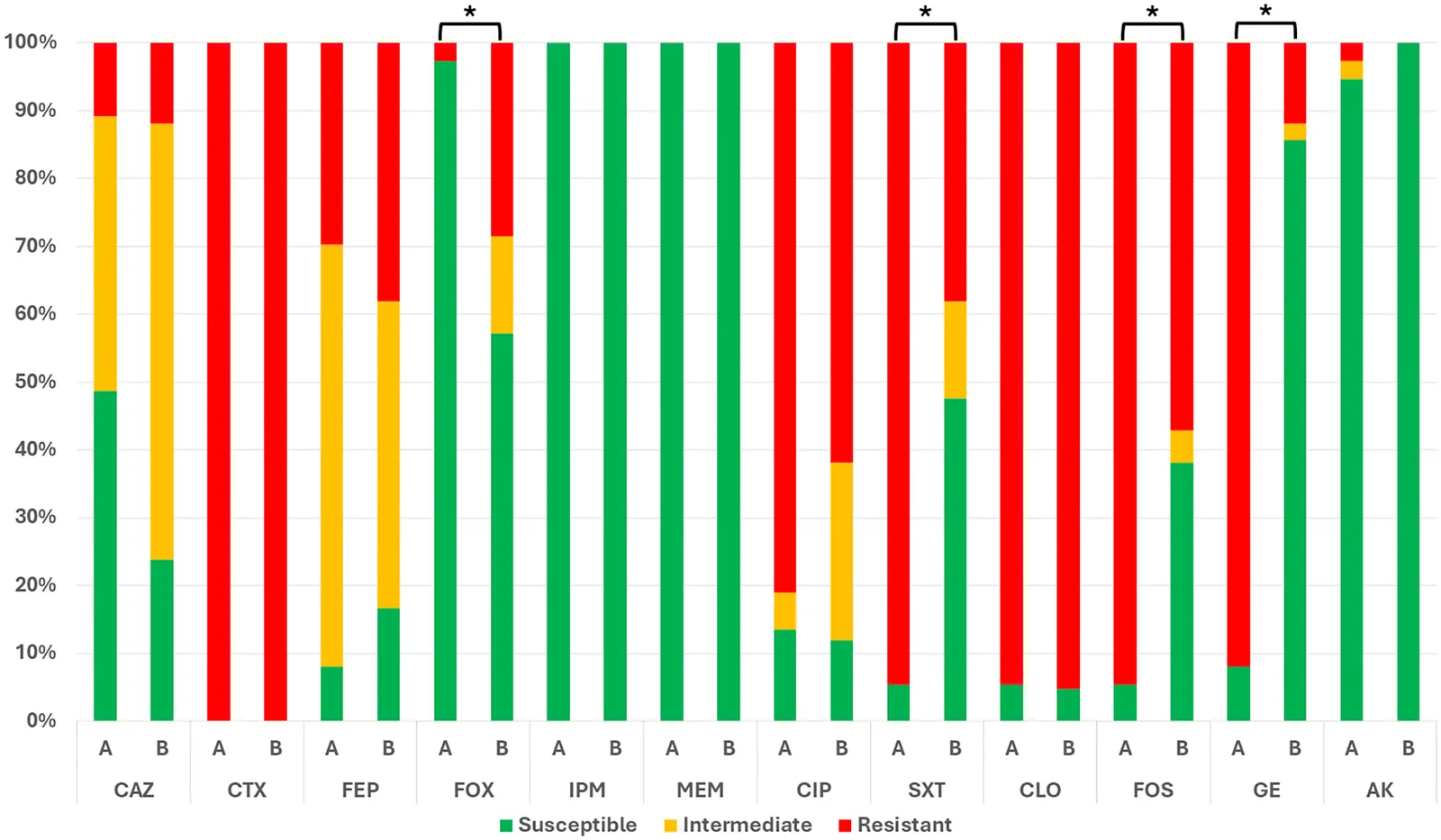
Susceptibility profile of the 79 ESBL-producing *E. coli* isolates recovered from cloacal swabs of chickens. CAZ: ceftazidime, CTX: cefotaxime, FEP: cefepime, FOX: cefoxitin, IPM: imipenem, MEM: meropenem, CIP: ciprofloxacin, SXT: trimethoprim/sulfamethoxazole, CHL: chloramphenicol, FOS: fosfomycin, GEN: gentamicin, AMK: amikacin. *: p < 0.05.

Additionally, high resistance rates (>60%) were observed for chloramphenicol (75/79; 95%), fosfomycin (59/79; 75%), ciprofloxacin (46/79; 71%), and trimethoprim–sulfamethoxazole (51/79; 65%), while resistance to gentamicin approached 50% (39/79). In contrast, amikacin showed a very low resistance rate (1/79; 1%), and all isolates remained susceptible to carbapenems.

Statistically significant differences (p < 0.05) in non-susceptibility frequencies between the two collection centers were observed for several antibiotics, including trimethoprim–sulfamethoxazole (A: 35/37; 95% vs. B: 22/42; 52%), fosfomycin (A: 35/37; 95% vs. B: 26/42; 62%), gentamicin (A: 34/37; 92% vs. B: 6/42; 14%), and cefoxitin (A: 1/37; 3% vs. B: 18/42; 43%).

### Antimicrobial resistance genes

Detection of CTX-M variants by qPCR revealed that 69/79 (87.3%; A:33/37, B:36/42) of isolates carried *bla*_CTX-M group 1_, while 10/79 (12.7%; A:4/37, B:6/42) carried *bla*_CTX-M group 9_, consistent with the phenotypic ESBL profiles. Whole-genome sequencing (WGS) analysis of nine representative ESBL-producing *E. coli* isolates identified key resistance determinants, which are summarized in Fig 2.

**Fig 2:**
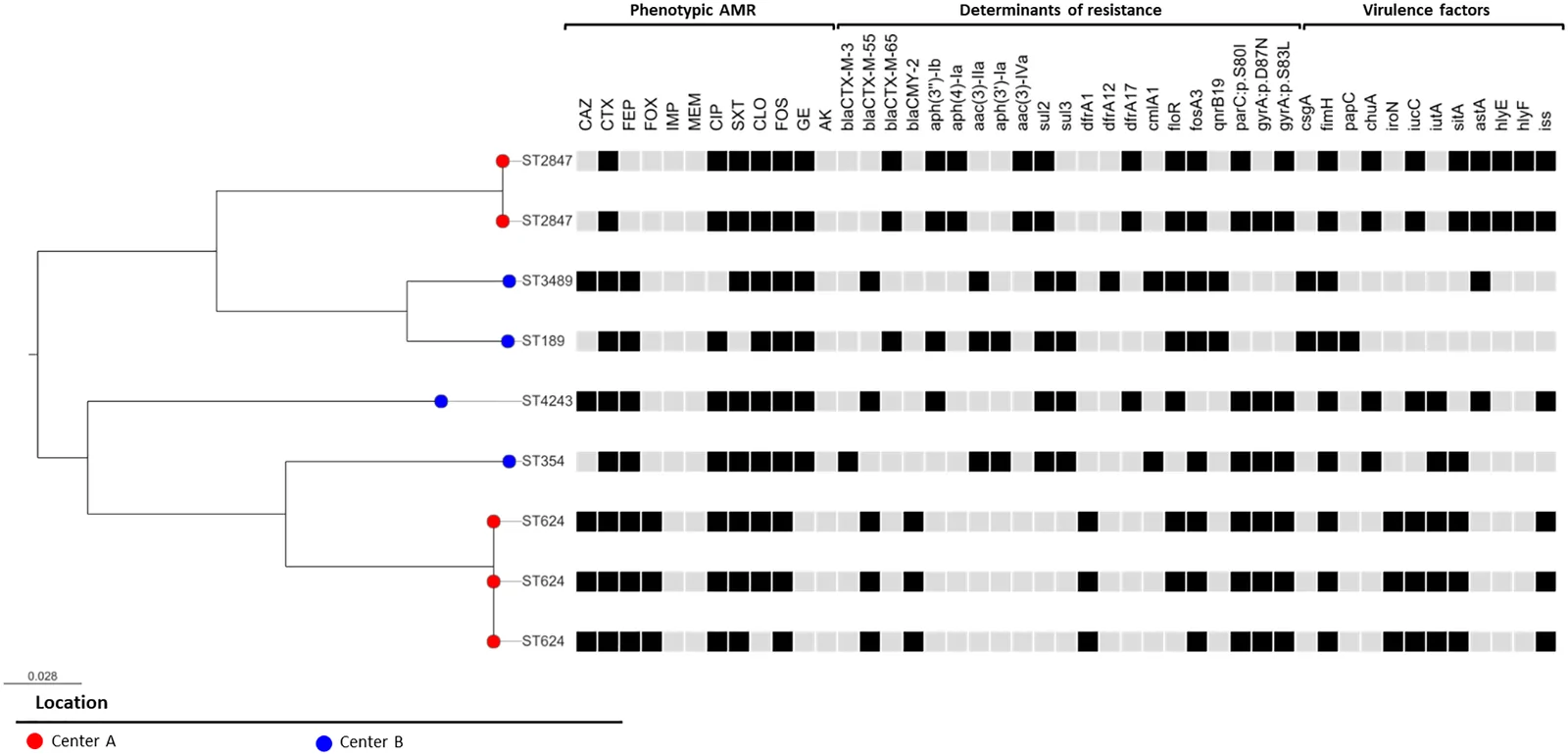
Phenotypic and genotypic characterization dendrogram of the 9 ESBL-producing *E. coli* isolates that underwent WGS. Details the phenotypic resistance, genotypic resistance and virulence factors as well as phylogeny of the collected ESBL *E. coli* isolates. The main determinants of resistance are summarized by bacterial isolate. ST: sequence type. CAZ: ceftazidime, CTX: cefotaxime, FEP: cefepime, FOX: cefoxitin, IPM: imipenem, MEM: meropenem, CIP: ciprofloxacin, SXT: trimethoprim/sulfamethoxazole, CHL: chloramphenicol, FOS: fosfomycin, GEN: gentamicin, AMK: amikacin.

### Phylogenetic groups and the virulence genes distribution

Phylogenetic classification of the sequenced isolates revealed membership in phylogenetic groups F (4/9), A (2/9), E (2/9), and D (1/9). Analysis of virulence-and adhesion-associated genes showed the presence of determinants involved in adhesion (*csg*, *fimH*, *papC*), iron and metal uptake (*chuA*, *iroN*, *iucC*, *iutA*, *sitA*), cytotoxicity (*astA*, *hlyE*, *hlyF*), and serum resistance (*iss*) (Fig 2).

### Genetic relationship

Maximum-likelihood phylogenetic analysis based on core-genome SNPs revealed the genetic relationships among the nine ESBL-producing *E. coli* isolates and their associated antimicrobial resistance determinants. The phylogeny demonstrated that the isolates clustered into two major deep branches (Fig 2). One branch comprised two isolates from the center B and two from the center A, while the second branch included two isolates from the center B and three from the center A.

Isolates from center A were genetically unrelated, showing divergence values ranging from 20,682–76,087 SNPs. In contrast, isolates from the center B formed two highly related clonal clusters: one cluster including isolates N44 and N7 (0 SNP differences), and a second cluster comprising isolates N30, N58, and N92, differing by 0–2 SNPs, indicating clonal relatedness.

## Discussion

AMR is widely recognized as a major global public health concern [3]. In the present study, 26.3% of isolates were identified as ESBL-producing *E. coli*, all of which exhibited MDR phenotypes. These findings are consistent with previous reports from Peru and other regions, suggesting that poultry may serve as a relevant reservoir of antimicrobial-resistant bacteria.

High resistance rates were observed across multiple antimicrobial classes, including fluoroquinolones, aminoglycosides, and sulfonamides, consistent with the widespread use of these antimicrobial families—such as ciprofloxacin and enrofloxacin, gentamicin and streptomycin, and cotrimoxazole—in meat production systems globally [22–24]. These patterns may reflect antimicrobial use practices in animal production systems; however, no direct data on antimicrobial use were available in this study. Therefore, any association between antimicrobial use and resistance patterns should be interpreted with caution.

Significant differences (p < 0.05) in antimicrobial susceptibility profiles were observed between the two collection centers. While these differences may suggest variability in antimicrobial exposure or management practices, the lack of farm-level data and the use of a convenience sampling approach significantly limit further interpretation.

Previous studies conducted in Peru support the high prevalence of MDR ESBL-producing *E. coli* in poultry, supporting the findings of the present study. Huamán *et al*. [12] reported that 85.5% of ESBL-producing *E. coli* isolates from 200 cloacal swabs collected between 2015 and 2016 exhibited high levels of antimicrobial resistance. Similarly, Ruiz-Roldán *et al*. [11] identified ESBL-producing *E. coli* in 59.4% of 32 chicken meat samples. At the international level, variability in resistance patterns, particularly for fosfomycin, has been described across different regions. In China, 27.4% fosfomycin resistance was reported among 234 ESBL-producing *E. coli* isolates recovered from chickens between 2014 and 2016 [25], a prevalence notably lower than that observed in the present study. In Latin America, reported fosfomycin resistance rates range from 5% to 40% in Brazil [26]. Likewise, Ortega-Paredes *et al*. [27] reported fosfomycin resistance rates of 50% in isolates obtained from chickens, sampled between 2017 and 2018 in Ecuador. These findings highlight the influence of local practices and epidemiological contexts.

Molecular analysis revealed a predominance of *bla*_CTX-M group 1_, consistent with previous studies in both animal and human settings [23,28,29]. Whole-genome sequencing of a subset of isolates identified *bla*_CTX-M-55_, *bla*_CTX-M-65_, and *bla*_CTX-M-3_ across multiple sequence types, suggesting the coexistence of genetic diversity alongside potential clonal dissemination. Similar diversity of CTX-M group 1 variants has been reported in poultry-associated isolates, as described by Parzygnat *et al*. [23]. Furthermore, the *bla*_CTX-M-65_ gene is also associated with ESBL-producing *Salmonella* Infantis isolates recently reported in Peru [30]. In addition, the detection of *bla*_CMY-2_ gene in isolates from one collection center was consistent with observed phenotypic resistance patterns, supporting the presence of AmpC β-lactamase activity [31].

A broad range of resistance genes associated with aminoglycosides, phenicols, folate pathway inhibitors, and fluoroquinolones was also identified, with strong concordance between genotypic and phenotypic resistance profiles. These findings highlight the potential role of commensal *E. coli* as a reservoir of transferable resistance determinants [32].

Most isolates belonged to phylogenetic group F, frequently reported in poultry-associated *E. coli* [33]. Notably, isolates from commensal-associated groups also carried virulence genes and ESBL determinants, highlighting the role of *E. coli* as a potential reservoir and vector of antimicrobial resistance [33,34]. Virulence gene analysis revealed the universal presence of *fimH* and the frequent detection of iron acquisition systems (*chuA, iroN, iucC, iutA, sitA*), suggesting a potential role in adhesion, colonization, and bacterial fitness [35,36].

Phylogenetic analysis suggested distinct patterns between sampling locations, with genetically diverse isolates in one center and clusters of closely related isolates in the other. While this may indicate differences in transmission dynamics, further studies incorporating farm-level epidemiological data are required to confirm these observations.

This study has several limitations. Sampling was conducted using a convenience approach at poultry collection centers, which may not fully represent farm-level dynamics. Additionally, no data on antimicrobial use or management practices were available, and whole-genome sequencing was limited to a small number of isolates. Therefore, the findings should be interpreted as preliminary and not generalized to all poultry production systems.

In conclusion, this study suggests that poultry production systems may serve as potential reservoirs of multidrug-resistant ESBL-producing *E. coli*. These findings support the need for further research and strengthened surveillance within a One Health framework to better understand the dynamics of antimicrobial resistance and to inform control strategies.
